# Development of rapid and highly accurate method to measure concentration of fibers in atmosphere using artificial intelligence and scanning electron microscopy

**DOI:** 10.1002/1348-9585.12238

**Published:** 2021-06-13

**Authors:** Yukiko Iida, Kenji Watanabe, Yusuke Ominami, Toshiyuki Toyoguchi, Takehiko Murayama, Masatoshi Honda

**Affiliations:** ^1^ Asbestos Risk Management Environmental Control Center Corporation Hachioji Tokyo Japan; ^2^ School of Environment and Society Tokyo Institute of Technology Yokohama Kanagawa Japan; ^3^ Innovation Division Hitachi High‐Tech Corporation Minato‐ku Tokyo Japan; ^4^ National Environmental Research and Training Institute Ministry of the Environment Government of Japan Tokorozawa Saitama Japan

**Keywords:** artificial intelligence, asbestos, fiber, image recognition system, scanning electron microscopy

## Abstract

**Aim:**

We aimed to develop a measurement method that can count fibers rapidly by scanning electron microscopy equipped with an artificial intelligence image recognition system (AI‐SEM), detecting thin fibers which cannot be observed by a conventional phase contrast microscopy (PCM) method.

**Methods:**

We created a simulation sampling filter of airborne fibers using water‐filtered chrysotile (white asbestos). A total of 108 images was taken of the samples at a 5 kV accelerating voltage with 10 000X magnification scanning electron microscopy (SEM). Each of three expert analysts counted 108 images and created a model answer for fibers. We trained the artificial intelligence (AI) using 25 of the 108 images. After the training, the AI counted fibers in 108 images again.

**Results:**

There was a 12.1% difference between the AI counting results and the model answer. At 10 000X magnification, AI‐SEM can detect 87.9% of fibers with a diameter of 0.06‐3 μm, which is similar to a skilled analyst. Fibers with a diameter of 0.2 μm or less cannot be confirmed by phase‐contrast microscopy (PCM). When observing the same area in 300 images with 1500X magnification SEM—as listed in the Asbestos Monitoring Manual (Ministry of the Environment)—with 10 000X SEM, the expected analysis time required for the trained AI is 5 h, whereas the expected time required for observation by an analyst is 251 h.

**Conclusion:**

The AI‐SEM can count thin fibers with higher accuracy and more quickly than conventional methods by PCM and SEM.

## 1 INTRODUCTION

In 2006, the use of asbestos was banned in Japan. The total import volume from 1930 to 2005 was 9.88 million tons. However, 80% of imported asbestos is still present in buildings. The demolition of buildings containing asbestos is expected to continue until approximately 2055.^1^ A report by the Ministry of Land, Infrastructure and Transport, Japan, shows the various points of use of asbestos in buildings.^2^

In the general environment, the total fiber concentration is 0.5 fibers/L or less.^3^ However, according to the survey at the Great Hanshin‐Awaji Earthquake of 1995, some airborne asbestos concentrations near the boundary of the demolition site were recorded 20 fibers/L (chrysotile), 160 fibers/L (crocidolite), and 250 fibers/L (crocidolite).^4^, ^5^ The occupational exposure limits for asbestos recommended by Japan Society for Occupational Health are 150 fibers/L (Chrysotile) and 30 fibers/L (Amosite, Crocidolite).^6^ But the measurement results near the demolition site had higher concentrations than the occupational exposure limits.

Therefore, it is important to prevent workers from being exposed asbestos and to prevent the spread of asbestos into the general environment at the demolition‐repair work of asbestos‐containing buildings. The Japanese government recognizes that asbestos demolition‐repair work produces high risk to the health of related person when poor countermeasures are taken. To prevent workers' exposure to asbestos, the law requires that they wear respiratory protection equipment with proven protective performance.^7^ And to prevent the spread of asbestos into the general environment, the demolition workplace has to be sealed with negative pressure. Air in the demolition workplace is first filtered through a high efficiency particulate air filter and then allowed in the open air. In addition, on‐site inspections are conducted by the local government to prevent asbestos dispersal during building demolition. However, some issues remain.

Issue 1: Measuring the concentration of airborne fibers typically requires 2‐3 days. In many cases, demolition works would have already been completed before the measurement results are obtained,^8^ which means that some on‐site inspection results are not used to prevent asbestos leakage.
Issue 2: It has been reported that asbestos fibers with a particle width thinner than 0.2 µm may result in the development of mesothelioma.^9^

When conducting fibers count with phase‐contrast microscopy (PCM), an objective lens with a numerical aperture of 0.75 is used for a phase‐contrast microscope, and the sample is irradiated with visible light.^10^ The theoretical resolution of a phase‐contrast microscope is 0.25 µm, assuming that the wavelength of visible light is 550 nm. PCM cannot count fibers smaller than 0.25 µm. We count fibers smaller than 3 µm in diameter and larger than 5 µm in length with aspect ratio (ratio of length to width) larger than 3 by PCM as the first screening round in the conventional airborne asbestos concentration measurement method. However, the number of fibers with 0.25 μm or more diameter and fibers with less than 0.25 μm diameter in the atmosphere is not proportional.^11^ Consequently, PCM cannot be used as indicators to monitor leaks of fibers of thinner than 0.2 μm in width. Observing fibers thinner than 0.2 μm is possible with electron microscopy. Using SEM equipped with an energy dispersive X‐ray (EDX) detector, if component analysis is required, it can be performed continuously even after fiber morphology analysis without changing the measuring instrument. Recent SEM using computer controlled sample stage is possible to return to any field previously viewed and can analyze the fiber components by EDX analysis, but the present study did not analyze the fiber component. Anyway, the fiber counting by electron microscopy is time‐consuming than that by PCM.^3^

Image analysis research using AI is being conducted in various fields for the purpose of quick and accurate diagnosis.^12^ The application of AI is advancing in all fields, and it has become possible to automate and increase the speed of image analysis work using AI, which was difficult previously. To improve the two issues mentioned, we attempted to apply SEM image analysis using AI to the analysis of fibers of 0.06‐3 μm in width and aspect ratio larger than 3 to realize a more rapid and highly accurate measurement.

## 2 OUTLINE OF RESEARCH

**FIGURE 1:**
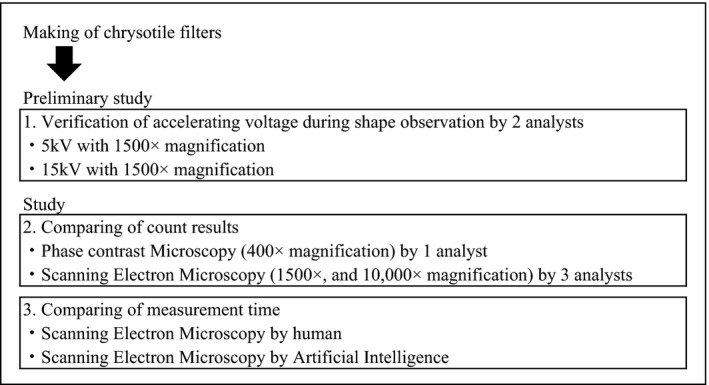
Outline of research

Figure 1 shows an outline of the research. After making filters filtrated chrysotile (chrysotile filters), we counted fibers using PCM and SEM methods. Then, we compared the measurement times of human‐ and AI‐performed SEM.

## 3 MATERIAL AND METHOD

### 3.1 Material

- SEM: *Miniscope TM4000* (Hitachi High‐Tech Corp.); chamber pressure: 1 Pa; observation: backscattered electron image.
- Accelerating voltage: 5 kV.
- PCM: *Eclipse 80i* (Nikon Corp.).

### 3.2 Method

**FIGURE 2:**
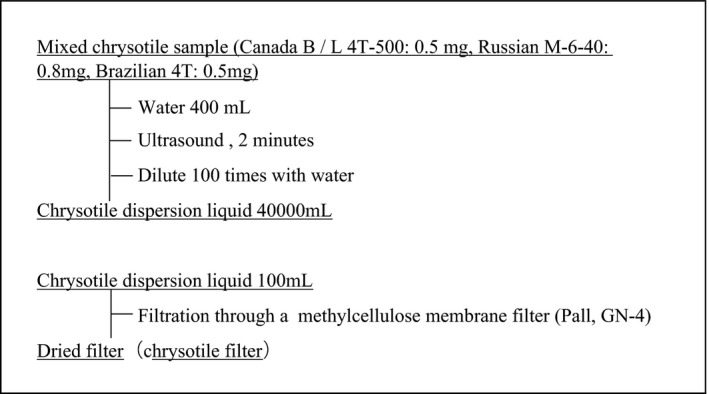
Preparation flow of chrysotile filters

Figure 2 shows the preparation flow of the chrysotile standard filter sample. In this study, we created a simulation sampling filter of airborne fibers using water‐filtered chrysotile (white asbestos). Chrysotile is often used in building materials. Chrysotile standard filter samples were prepared by mixing chrysotile from three production areas to obtain standard data. Canada B/L 4T‐500:0.5 mg, Soviet Union M‐6‐40: 0.8 mg, and Brazilian 4T: 0.5 mg were mixed in a beaker; 400 ml of distilled water was added to the beaker, and the contents were ultrasonically irradiated for 2 min. Immediately thereafter, chrysotile‐dispersed water was diluted 100‐fold to prepare a test solution. The test solution was filtered through a methylcellulose membrane filter (Pall Corp., GN‐4) with a pore size of 0.8 μm and a diameter of 47 mm; then, the filter was dried. The dried chrysotile standard filter sample was divided into four, and one piece was used to prepare a chrysotile standard slide for PCM by the acetone‐triacetin method according to the Asbestos Monitoring Manual.^4^ Another piece cut out from the remaining filter sample to a size of approximately 5 × 5 mm was fixed to a brass sample stage with a conductive carbon double‐sided tape, this was used as a chrysotile standard sample for SEM. This sample was not coated with a conductive material on the sample surface.

An *Eclipse 80i* (Nikon Corp.) phase‐contrast microscope was used to observe the chrysotile standard slide. An objective lens with 40X magnification (positive contrast, numerical aperture 0.75) and an eyepiece lens with 10X magnification (with an eyepiece graticule in the form of a circle of diameter 300 μm attached) were attached to a PCM.

**FIGURE 3:**
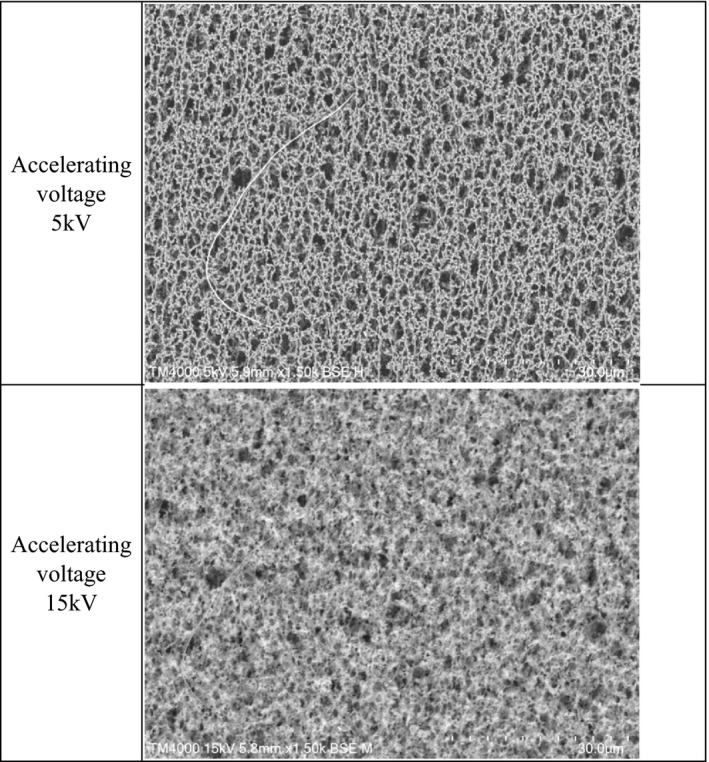
Same field‐of‐view image at 5 and 15 kV accelerating voltage scanning electron microscopy (SEM 1500X)

The SEM used in this study was *FlexSEM1000Ⅱ* (Hitachi High‐Tech Corp.). The chrysotile standard sample was introduced into the SEM, and the backscattered electron image of the sample was observed in a low‐vacuum mode (Chamber pressure: 1 Pa). The backscattered electron image was observed because the secondary electron image of the sample could not be observed in the low‐vacuum mode. It is known that when a thin sample is irradiated with electrons at an accelerating voltage that is very high, secondary electrons generated on the back side of the sample interfere with each other, and a clear backscattered electron image cannot be obtained. In the preliminary study of this research, it was found that the backscattered electron image became unclear at an accelerating voltage of 15 kV and was clearest at an accelerating voltage of 5 kV (Figure 3).

The wavelength of the electron beam used in the electron microscope was approximately 0.017 nm at an accelerating voltage of 5 kV. In low‐vacuum observation, the electron beam irradiated on the sample is scattered by the residual gas molecules in the sample chamber; hence, it is difficult to calculate the resolution. In this study, the fiber width was measured manually by magnifying the acquired SEM images. Automated measurement using image analysis software remains a future work. Fibers with a width of 0.06 μm or more were confirmed in the images. However, because the pixel size of the acquired images is approximately 0.01 μm, the measurement error expected from the pixel size of the image is ±0.02 μm. The minimum fiber width that can be observed on the screen by eye‐sight considering the single measurement error is approximately 0.06 ± 0.02 μm. The statistical measurement error is expected to decrease as the number of fibers increases.

A total of 108 images of field was taken at a 5 kV accelerating voltage with 10 000X magnification SEM. Three experts were chosen to manually count the samples. Each of the three analysts counted 108 images and created a model answer for fibers. Artificial intelligence (AI) image recognition was performed with commercial deep learning software (ViDi Suite Version 3.2, Cognex). Deep learning is a type of machine learning consisting of artificial deep neural networks to train computers to solve cognitive tasks such as natural language processing and image recognition.^13^ The software was originally developed for industrial image analysis to classify anomalies in images^14^, ^15^ and is currently also used to analyze medical images.^16^, ^17^ In this study, a supervised ViDi detection tool was used to detect fibers in the SEM images. First, 25 of the 108 acquired images were used as the training data set, for which the fibers in the images were annotated and analyzed to train the deep learning model. Then, the entire data set was used to validate the model after training. The analysts counted fibers detected by AI, based on the generated segmentation maps.

**FIGURE 4:**
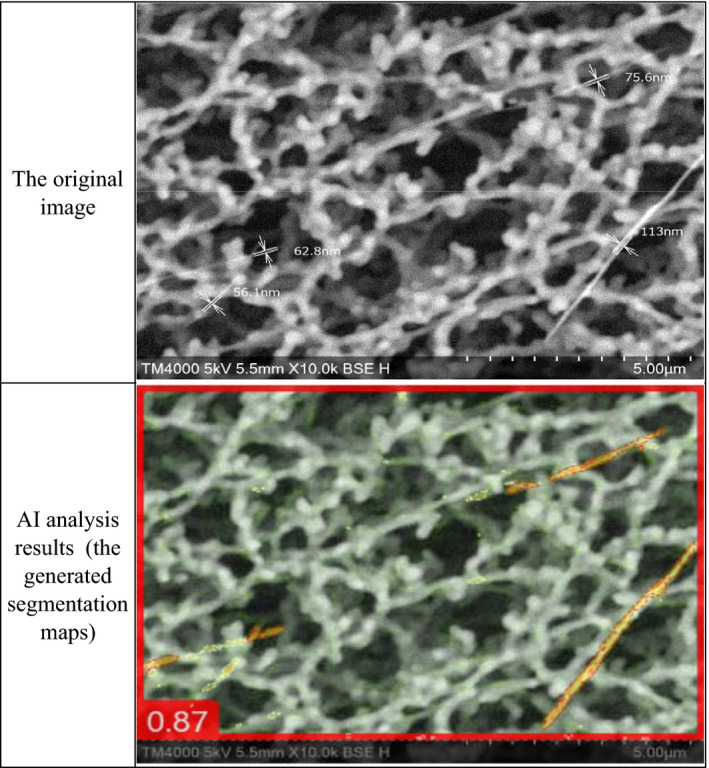
Original image and artificial intelligence (AI) analysis result of the same field of view scanning electron microscopy (SEM 10 000X)

We compared the number of fibers in the SEM images counted by the analysts and the AI. The AI trained for fiber detection colored the particles of the SEM images it identified as fibers (Figure 4). In cases where the AI colored only some parts of the particles, we determined that the AI had found the whole fiber. In addition, we estimated the time required to observe the same area in 300 images with a 1500X magnification SEM listed in the Asbestos Monitoring Manual (Ministry of the Environment) and a 10 000X SEM.

## 4 RESULTS

### 4.1 Comparison of measurement time of SEM and AI‐SEM

We compared the time required for measurement by SEM and AI‐SEM. The time required for an analyst to count 108 SEM images at 10 000X magnification was 2 h 30 min. The time required for an analyst to count one image was 1.4 min. The AI‐SEM took 3 min to analyze 108 SEM images at 10 000X magnification. The time required to analyze one image by AI was 0.03 min. When observing the same area in 300 images with the 1500X magnification—as SEM listed in the Asbestos Monitoring Manual (Ministry of the Environment)—by the 10 000X SEM, the expected measurement time required for the trained AI is 5.4 h, and the expected time required for observation by an analyst is 251 h.

AI‐SEM was used to analyze fibers with a fiber width of 0.06‐3 µm. The analysis was performed at 10 000X magnification. The time required for the 10 000X AI‐SEM analysis was approximately twice that of SEM by an analyst using the conventional method at 1500X magnification. A 245 h reduction was expected in the 10 000X AI‐SEM analysis compared with the human count at the same magnification SEM.

### 4.2 Ability of AI image recognition system to detect fibers in SEM images

The theoretical resolution of the PCM in this study is 0.25 μm. The SEM used in this study measured the particle width at 10 000X magnification and confirmed that particles of 0.06 μm and larger could be observed. Therefore, it is possible to observe fibers thinner than 0.2 μm with the SEM of this study. Table 1 shows the results of counting fibers from the same filter sample using PCM at 400X magnification, SEM at 1500X magnification, and SEM at 10 000X magnification. Table 1 shows the results of an analyst counting individual fibers with fiber widths of 0.06‐0.1, 0.1‐0.2, and 0.2 μm and more using SEM at 10 000X magnification. As expected, the SEM count results contain numerous thin fibers that could not be counted by PCM. It has been reported that even in actual environments, asbestos may be confirmed by electron microscopy despite asbestos‐like particles not being confirmed by PCM.^11^

**TABLE 1:** The count results by phase contrast microscopy (PCM) and scanning electron microscopy (SEM) with the same filter

Microscopy	Magnification	Observation area/view (μm^2^)	Analyst	Fibers/field of views	Estimated number of fibers per field of a view of PCM (fibers/70 650 μm^2^)	Diameter of the fibers (μm)
PCM	400	70 650	A	200/32	6	0.25 μm≦Diameter of the fibers ≦3 μm
SEM	1500	5378	A	167/38	58	0.2 μm≦Diameter of the fibers ≦3 μm
			B	121/38	42	
	10 000	150	A	103/108 (0.06‐0.1 μm: 75, 0.1‐0.2 μm: 27, 0. 2 μm or more: 1)	449 (0.06‐ 0.1 μm: 327, 0.1‐0.2 μm: 118, 0.2 μm or more: 4)	0.06 μm ≦ Diameter of the fibers ≦3 μm
			B	108/108	471	
			C	92/108	401	

The measurement results of the analysts and AI were compared (Table 2). The analyst and AI measurements were 87.9% in agreement. The difference between the AI and analyst counting results is 12.1%. The reason for analyst detection but not AI detection was that the AI false negative the “thinner fibers.” In addition, false detections confirmed that the AI counted fibers that were not counted by humans. The main reason for the false detection was that the AI counted fibers of the sampling filter as fibers. It is expected that the false negative and false detection can be improved by increasing the amount of training for the AI.

**TABLE 2:** The measurement results of the analysts and the artificial intelligence (AI)

		Number
A	Total number of fibers (=B + C + D)	132
B	The number of fibers that both analysts and AI were able to detect	112
C	The number of fibers that detected by analysts, but overlooked by AI (thin fiber overlooks by AI: 16)	16
D	Number of fibers that overlooked by analysts, but detected by AI (thin fiber overlooks by analysts: 3)	4
E	AI detected particles with a shape that does not count as fibers (AI detected fibers of filter as fibers: 32)	36
		Percentage (%)
(B + D)/A	AI detection rate (%)	87.9
C/A	AI false negative rate (%)	12.1
E/(A + E)	AI false detection rate (%)	21.4

## 5 DISCUSSION

In this study, AI exhibited a 12.1% false negative and a 21.4% false detection. However, it has been reported that PCM has an error of ±20%, even for a well‐trained analyst. Microscopy is an analytical method in which a “human” act as a detector. Therefore, often, there is an “error” in the judgment owing to the difference in the training level of the analyst and the difference in the count ability. If a well‐trained AI takes the role of SEM detector instead of humans, it is expected to detect fiber with less error than the humans. In this study, 25 images were used as the training data set for AI. The number of images used for AI training was not large. It is expected that the false negative rate and the false detection rate will decrease if the amount of training for AI is increased.

A methylcellulose membrane filter with a pore size of 0.8 μm (Pall Corp., GN‐4) was used in this study. This type of filter can be used for both PCM and SEM measurements; therefore, we could compare the measurement results of PCM and SEM using the same filter. But the sponge shape of methylcellulose membrane filters resembled fibers; therefore, AI counted fibers of the sampling filter as fibers. In the next stage of the study, we plan to use a polycarbonate filter (filter cord: millipore 0.8 mm ATTP) dedicated to electron microscopy. The filter has a round hole shot through the filter with an electron gun; we expect that it is unlikely that the filter will look fibrous.

### 5.1 Future study

The pixel‐wise accuracy of segmentation by the AI requires improvement. For example, there were falsely detected pixels in the filter and undetected pixels in fibers with twisting, distortion, branches, or particles (Figure 4, generated segmentation maps). Precise contour extraction of fibers remains a future work to automate quantitative analyses such as counting the number of fibers or measuring the length or width of fibers.

### 5.2 Limitations

In this research, we created a simulated air sampling filter of chrysotile using water‐filtered chrysotile. We have not verified it for filtering amosite, crocidolite, tremolite, or other fibers or dust.

## 6 CONCLUSION

The results showed that AI can detect fibers 50 times faster than humans. Regarding the analysis accuracy, a 12.1% false negative rate and 21.4% false detection were confirmed; improvement is needed in the next research step. As the first step of the research, AI‐SEM has shown the possibility of shortening the measurement time of the airborne fibers while maintaining high analysis accuracy. The results of this research are not sufficient for the development of AI‐SEM; it is necessary to continue to verify and validate it in the future. We would like to bring this measurement method to practical use and contribute to the prevention of environmental pollution caused by asbestos scattering from asbestos‐containing building demolition workplaces, which in turn would mitigate the ill‐effects of pollution on the health of local residents.

## DISCLOSURE

*Approval of the research protocol*: N/A. *Informed consent*: N/A. *Registry and the Registration No. of the study/trial*: N/A. *Animal studies*: N/A. *Conflict of interest*: Coauthors Kenji Watanabe and Yusuke Ominami are employees of Hitachi High‐Tech Corporation.

## Data Availability

​Data are available upon reasonable request.
